# Materials for Pharmaceutical Dosage Forms: Molecular Pharmaceutics and Controlled Release Drug Delivery Aspects

**DOI:** 10.3390/ijms11093298

**Published:** 2010-09-15

**Authors:** Heidi M. Mansour, MinJi Sohn, Abeer Al-Ghananeem, Patrick P. DeLuca

**Affiliations:** Division of Pharmaceutical Sciences, College of Pharmacy, University of Kentucky, Lexington, KY 40536, USA; E-Mails: minjisohn@uky.edu (M.S.); amalg0@email.uky.edu (A.A.-G.); ppdelu1@email.uky.edu (P.P.D)

**Keywords:** polymers, copolymers, biomaterials, biodegradable, microparticle, nanoparticle, pharmaceutical dosage forms, particle engineering design, manufacture

## Abstract

Controlled release delivery is available for many routes of administration and offers many advantages (as microparticles and nanoparticles) over immediate release delivery. These advantages include reduced dosing frequency, better therapeutic control, fewer side effects, and, consequently, these dosage forms are well accepted by patients. Advances in polymer material science, particle engineering design, manufacture, and nanotechnology have led the way to the introduction of several marketed controlled release products and several more are in pre-clinical and clinical development.

## 1. Introduction

Biodegradable and biocompatible materials for pharmaceutical dosage forms have enabled the advancement of pharmaceuticals by providing better therapy and disease state management for patients through controlled release drug delivery, particularly as microparticles and nanoparticles. Controlled release delivery is available for many routes of administration and offers many advantages over immediate release delivery. This review describes controlled drug delivery, the types/classes of biocompatible and biodegradable pharmaceutical polymers, the types of drugs encapsulated in pharmaceutical polymers, microparticle/nanoparticle controlled drug delivery, the particle engineering design technologies and manufacture of controlled release microparticles/nanoparticles, and currently approved controlled release drug products.

## 2. Controlled Drug Release Technology of Drugs

In order to achieve efficient disease management, the concentration of released drugs from polymeric matrices should be within the therapeutic window with minimal fluctuation in blood levels over prolonged periods of time at the intended site of action [1–3]. The release of drug can be controlled by diffusion, erosion, osmotic-mediated events or combinations of these mechanisms [4,5]. Typically, a triphasic release pattern is observed, consisting of an initial burst [4], primarily attributed to drug precipitates at the particle surface and surface pores in the polymer, and the osmotic forces in highly water-soluble peptide formulations [6], a lag period depending on the molecular weight and polymer end-capping [5] and finally erosion-accelerated release [6].

Considering release rate control as a key parameter, a decrease in particle size (*i.e.*, an increase in the specific surface area) results in higher release [6]. Also, higher porosity of the particles inducing a larger inner surface can increase the influx of the release medium into the particles and, thereby, facilitate the drug diffusion rate [7]. In addition, the specific properties of the polymer matrix (e.g., the chain length, flexibility and swelling behavior, potential interactions between polymer and drug) will significantly influence the drug release rate [8,9]. Therefore, switching to a different molecular weight or an end group capped polymer, and the use of block copolymers will alter the diffusion and drug release rate [10,11].

To achieve zero-order release kinetics indicative of uniform release with respect to time, which is desired for most applications, a combination of fast- and slow-releasing particles or the use of copolymers are possible alternative advanced methods [12,13]. A one-time only dose can be achieved by co-injection of a bolus of soluble drug as a loading dose and zero-order releasing microspheres as a maintenance dose.

## 3. Types and Classes of Biodegradable and Biocompatible Pharmaceutical Polymers

Biodegradability and biocompatibility of a polymer are among the most important properties for pharmaceutical applications. Biodegradation is generally described by two steps, namely: (1). water penetrates polymeric matrix, attacking the chemical bonds by hydrolysis and thereby shortening the polymer chain length resulting in a reduction in molecular weight and metabolism of the fragments and bulk erosion; and (2). surface erosion of the polymer occurs when the rate at which the water molecules penetrating the matrix is slower than the rate of conversion of the polymer into water-soluble materials. Biocompatibility refers to specific properties of a material not having toxic or injurious effects on biological systems. Non-biocompatible materials can cause irreversible tissue damage, such as permanent tissue destruction, necrosis, significant fibrosis, and dystrophic calcification.

However, it should be noted that good biocompatibility does not insure good biodegradability. Poly(*N*-isopropyl acrylamide) (NIPAAM), used to formulate thermo-responsive hydrogels [14], is non-toxic and biocompatible but not biodegradable by hydrolysis. It is of critical importance to investigate both biodegradability and biocompatibility of synthesized copolymers [15,16].

### 3.1. Polyester-Based Synthetic Polymers

**Figure 1:**
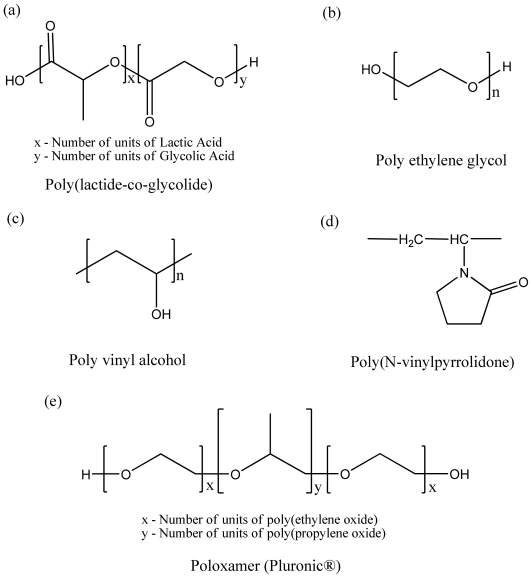
Structures of biodegradable and biocompatible polymers.

Drug delivery systems based on biodegradable aliphatic polyesters have advanced remarkably over the past few decades. Commonly used polymers (Figure 1 and Table 1) such as poly (ɛ-caprolactone) (PCL), poly (lactide acid) (PLA), and poly (lactic-co-glycolic acid) (PLGA) are FDA-approved and well known for their biodegradability, biocompatibility and non-toxic properties which makes them suitable as matrices for controlled release drug delivery systems. Poly (lactic-co-glycolic acid) (PLGA) has become one of the most studied diblock copolymer biomaterials for drug encapsulation and is present in several commercially available pharmaceutical products. Due to the slow degradation and drug release rates, poly (lactide acid) (PLA) homopolymer has no longer been broadly used for the past two decades. PLGA heteropolymer degrades relatively faster than PLA and can achieve 2–6 weeks release criteria, while PLA delivers drugs over months [7].

Degradation rate of PLGA is attributed to its molecular weight, its distribution, the lactide/glycolide ratio, the polymer end-group, micro/nano particle size, pH, and the temperature of the release medium. Generally, low molecular weight of PLGA degrades faster and as a result it causes more rapid drug release and higher initial burst [17]. The hydrophilicity of PLGA is defined by the lactide:glycolide ratio and affects the release rate in a micro/nano particulate formulation. When the lactide/glycolide ratio increases, the drug release rate decreases [18]. The carboxyl end groups of PLGA are mainly involved in interactions with drug. The initial adsorption of a peptide to hydrophilic PLGA is due to an ionic interaction between the amino group of the peptide and the terminal carboxyl group of PLGA, resulting in initial burst release [10,11]. Also, hydrophilic and acidic properties of free carboxyl groups induce faster water uptake and hydrolysis of ester bonds making more acidic groups by autocatalytic cycle [19]. For particulate drug delivery systems, particle preparation procedure and interactions of polymer with drug, both play important roles in polymer degradation. Higher stirring rate [20] and ultrasound treatment [6] for emulsification can reduce particle size (thereby increasing the surface area per unit volume) of particles resulting in faster degradation upon exposure to the release medium.

In comparison to PLGA, Poly (ɛ-caprolactone) (PCL) has high permeability to small drug molecules and a slow degradation rate which make it suitable for extended long-term delivery over a period of more than a year. While PLGA generates an acidic environment during degradation, which can lead to peptide/protein instability, the ability to avoid acidic conditions has become one of major advantages for selecting PCL as a drug carrier [21].

Poly(ethylene glycol) (PEG), also known as polyethylene oxide (PEO), is a largely exploited polymer for advanced physical and chemical stability of drugs and its “stealth” properties. Abuchowski, Davis and co-workers first described a method for the covalent attachment of mPEG to proteins in 1977 [22], which has since been termed PEGylation. PEG is an amphiphilic polymer composed of repeating ethylene oxide subunits and can dissolve in organic solvents as well as in water. Ordinarily, the properties of PEG that are of particular relevance in pharmaceutical applications are: (1). improved circulation time due to evasion for renal or cellular clearance mechanisms; (2). reduced antigenicity and escape from phagocytosis and proteolysis; (3). improved solubility and stability; and (4). reduced dosage frequency, with reduced toxicity [10,23,24]. Degradation rate of PEG depends both on the molecular weight and on the concentration of PEG. The degradation mechanism is explained by the strong hydrophilicity of PEG, the hydrogen-bonding interaction between PEG and water [23].

Polyvinyl alcohol (PVA), a homopolymer with measurable surface activity, has some similarities with PEG in that it is comprised of a repeating monomer unit that is hydrophilic, as shown in Figure 1. PVA plays a variety of functions in controlled release delivery systems including the following; as a matrix of particle [25], hydrogel [26], and as a surfactant in emulsion systems during formulation processes for micro/nano particles [27–29]. PVA can be grafted with a chain of polymeric substrate [30,31]. For example, in PVA-grafted PLGA polymer, the PVA backbone can be modified to create negatively or positively charged properties using sulfobutyl or amine moieties and the resulting increase in the hydrophilicity of this polymer provides advantages when carrying sensitive biomolecules, such as proteins, peptides and DNA [30].

As shown in Figure 1 and Table 1, poly(*N*-vinylpyrrolidone) (PVP) has been extensively used in controlled release drug delivery due to its biocompatibility, chemical stability, and excellent aqueous solubility [24,32]. Moreover, a polymer matrix combined with PVP has been known to reduce nonspecific protein adsorption [33]. Kollidon^®^SR is a compressible polymeric blend composed of polyvinyl acetate (PVAc) and povidone (PVP) commercially available and used often in pharmaceutical dosage forms [34]. The amorphous nature of PVAc and its low glass temperature (T_g_) of 28–31 °C impart unique characteristics to Kollidon^®^SR. By the gradual leaching of water-soluble PVP, the matrix creates channels for releasing drugs [35]. Due to excellent solubility, the soluble grades of Kollidon^®^ usually have no delaying effects on the dissolution of drugs and can be used as a hydrophilic component in dosage forms that contain controlled-release excipients, such as cetylalcohol, alginate, cellulose derivatives, polyactic acid, polyvinyl alcohol, ceresine wax, stearic acid or methacylate copolymers to control the release of drugs, as binders or sometimes as plasticizers [34].

### 3.2. Natural Origin Polymers Used as Pharmaceutical Excipients

Naturally derived polymers with special focus on polysaccharides and proteins have become attractive in the biological applications of controlled release systems due to their similarities with the extracellular matrix in the human body and favorable specific properties that can be exploited for “smart” systems, for example, stimuli-responsiveness. Polysaccharides are a class of biopolymers constituted by either of one or two alternating monosaccharides, which differ in their monosaccharide units in the length of a chain, in the types of the linking units and in the degree of branching [36]. Table 1 lists FDA-approved natural origin polymers and their routes of administration.

Starch, composed of amylose and amylopectin, is generally modified to change its physical properties by adding plasticizers, such as water and glycerol, improving the flexibility of starch which is favorable in pharmaceutical applications [37,38]. In addition, cross-linking techniques can lead to advanced drug delivery systems by compensating for weak points of plasticized starch which is sensitive to moisture, shows low tensile strength and Young’s modulus [39]. Due to its high hydrophilicity, starch has bioadhesive properties [8] that are favorable for ophthalmologic drug delivery (*i.e.*, timolol, flurbiprofen) [40].

Chitosan is a polyaminosaccharide, prepared by the N-deacetylation of chitin. Chitosan is thermo-stable due to its strong intramolecular hydrogen bonding between hydroxyl and amino groups. As a weak poly-base, reversible pH-sensitive behavior, due to its large quantities of amino groups on its chain, makes chitosan applicable in hydrogel smart delivery systems. Chitosan is soluble in water and in organic acids such as formic, tartaric, acetic, and citric at low pH (<pH 6.5) due to protonation of the amino groups [41]. For particulate drug delivery, a cross-linking technique by glutaraldehyde is generally used [42].

Alginate, a marine-derived polysaccharide, is abundantly available in nature and is an attractive alternative for controlled release systems, as it is amenable to sterilization and storage [43]. Alginate is an anionic block copolymer consisting of β-d-mannuronic acid (M) and α-L-guluronic acid (G).

Alginate forms a stimuli responsive hydrogel in two different ways. One is via hydrogen bonding at pH levels below 2, which is based on the pKa values for carboxyl acid groups in M (pKa 3.38) and G (pKa 3.65). The other way is via ionic interactions with divalent metal ions. Since, chelating agents such as EDTA or phosphate buffer can easily remove Ca^2+^ ions, Ca^2+^-responsive-hydrogel systems can be designed [44].

Hyaluronic acid is a major carbohydrate component of the extracellular matrix found in synovial fluids and on cartilage surfaces [45]. Hyaluronic acid, an excellent lubricator and shock absorber, inhibits chondrocytic chondrolysis, thereby improving the lubrication of surfaces and reducing joint pain in osteoarthritis [46]. Hyaluronic acid has been widely studied for drug delivery, especially for transplantation, injection and gene delivery particularly as it is non-immunological [45]. To avoid rapid degradation and clearance, when the hyaluronic acid is used as a carrier, its matrix is utilized with cross-linking using glurataldehyde [37], carbodiimide [37], or polyethyleneglycol diglycidylether (PEGDG) [46].

Bovine Serum Albumin (BSA), a globular protein, is a naturally biodegradable, nontoxic and non-antigenic biopolymer making it suitable for controlled drug delivery. Typically BSA particles are prepared under mild conditions by coacervation or a desolvation process [47,48] and cross-linked by glutaraldehyde. However, polyethyleneimine (PEI) has been suggested to avoid potential toxicity of glutaraldehyde [49].

Collagen is the major protein component of the extracellular matrix. Twenty seven types of collagens have been identified to date, but collagen type I is the most investigated for pharmaceutical applications [37]. Several factors affect degradability of collagen, for example, structure contraction caused by cell penetration, collagenase, gelatinase and other non-specific proteinases can digest collagen [50]. The versatile properties of collagen (e.g., high mechanical strength, good biocompatibility, low antigenicity, and water uptake properties) have made it one of the most useful biomaterials for tissue engineering using a form of collagen sponge [51] or collagen gel [52].

Gelatin is a denatured protein obtained by acid and alkaline processing of collagen [37]. Gelatin, in a variety of isoelectric points, can be manufactured and basic gelatin with an isoelectic point of 9.0 and acidic gelatin with an isoelectric pont of 5.0 are mostly used. If the biomolecule to be released is acidic, basic gelatin with an isoelectric point of 9.0 is preferable as a matrix, and *vice versa*. Both gelatins are insoluble in water. To prepare a hydrogel through cross-liking, the gelatin hydrogels forming polyion complexes with proteins will facilitate the release of biologically active proteins [53].

### 3.3. Homo vs. Diblock Copolymer vs. Triblock Copolymers

To enhance the desirable properties of polymer as a matrix for a controlled drug delivery system, efforts have been made to improve its hydrophilicity, biodegradation rate, and drug stability. The most commonly used hydrophilic block for polymeric drug delivery systems is poly (ethylene oxide)/poly(ethylene glycol), PEO/PEG. PEO is FDA-approved for parenteral administration, due to its low toxicity and biocompatibility [10]. One of the primary advantages of attachment of the PEO moiety is its effectiveness against protein adsorption to hydrophobic surfaces. For polymeric micelles, the length of the PEO blocks affects circulation time and uptake by phagocytes, with longer chains extending circulation time and reducing phagocytosis [54]. As a shell forming material for polymeric micelles, with PEO, PEG (Figure 1) imparts to the micelle with a “stealth character” in the blood compartment, achieving longer circulation [55]. PEG grafted to surfaces of nanospheres proved to reduce thrombogenicity and to increase their dispersion stability in aqueous medium, due to steric repulsion effects of tethered PEG strands [56]. PLGA-PEG-PLGA (ReGel) as controlled release formulations for two weeks delivery of glucagon-like peptide-1 (GLP-1) in type 2 diabetic rats [16]. PEG-PLGA-PEG triblock copolymers with TGF-β1 have been formulated to accelerate the diabetic wound healing [57]. PVA based branched graft polyester bearing PLGA block, which is first generation, designated as PVA-*graft*-PLGA, shows lower burst effects and controlled release profiles based on the structure and molecular weight of the copolymer [58]. In order to obtain negative charged polymer, as a second generation, branched poly[sulfobutyl-poly(vinyl alcohol)-*g*-(lactide-*co-*glycolide)] (SB-PVA-*g*-PLGA) was reported in which the sulfobutyl groups are covalently conjugated to PVA backbone [59,60]. Third generation, amine-PVA-*g*-PLGA, was developed by attaching various amino groups to the PVA backbone, which is positively charged [61].

Poloxamers, also known by the trade name Pluronics, are nonionic triblock copolymers composed of hydrophilic poly(ethylene oxide) (PEO) and hydrophobic poly(propylene oxide) (PPO) blocks, designated as PEO-PPO-PEO [62]. Due to their amphiphilic characteristics poloxamer exhibits surfactant properties coupled with ability to self-assemble into micelles above critical micelle concentration (CMC) in aqueous solutions. Besides, these copolymers are shown to be potent biological response modifiers capable of overcoming drug resistance in cancer and enhancing drug transport across cellular barriers, such as brain endothelium [63,64].

## 4. Therapeutic Agents Encapsulated in Polymeric Particles

**Figure 2:**
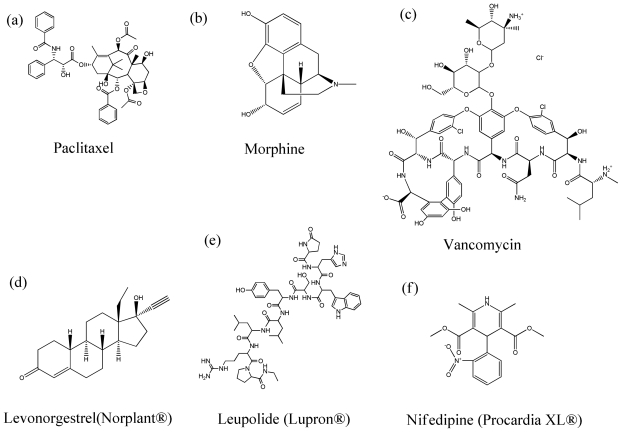
Various therapeutic agents from different therapeutic classes that have been encapsulated in polymeric particles.

Administration of a variety of drugs from different therapeutic classes encapsulated in polymeric particles (Figure 2), particularly through parenteral route, has been extensively investigated to lead to complete absorption of drugs in the systemic circulation and control drug release over a predetermined time span ranging from days to weeks to months.

In chemotherapy, obtaining adequate drug levels at the tumor cell is the most primary issue because inadequate tumor cell drug-burden will lead to low cell apoptosis and to early development of drug resistance [65]. Chemotherapeutic agents (Figure 2) such as paclitaxel [66–68], docetaxel [69], vascular endothelial growth factor siRNA [70,71], 5-fluorourasil [72,73], doxorubicin [74,75], adriamycin [76], gancyclovir [77], celecoxib [78,79], bleomycin [80,81], and tamoxifen [29] have been successfully formulated in polymeric particulate delivery systems.

In pain control, opioids (Figure 2) are vital in the treatment of severe and chronic pain associated with cancer and certain chronic diseases. Morphine [82,83], nalbuphine [84], tramadol [85], buprenorphine [86,87], fentanyl [88,89], and hydromorphone [90] have been developed to accomplish prolonged drug release so that patients’ compliances, and by extension, qualify of life for patients suffering chronic pain can be improved. In addition, non-steroidal anti-inflammatory drugs (NSAIDs) such as flubiprofen [91], ibuprofen [92], celecoxib [93,94], diclofenac [95,96], and indomethacin [97,98], have been developed as encapsulated drug microspheres. Several local anesthetics also have been reported with opioids [90,99]. Lidocaine [100], tetracaine [101], bupivacaine [90], and ropivacaine [102] were studied for drug encapsulated polymeric particulate system.

Antibiotic drug delivery will decrease the bacterial load at the infection site, minimizing renal, liver and systemic toxicities. Application of controlled drug release systems offers advantages in maintaining a highly site specific drug concentration for an extended period while reducing systemic toxicity and drug resistance. Antibiotics (Figure 2) incorporated in controlled release systems include chlorhexidine [103], vancomycin [104], amphotericin B [105], gentamicin [106,107], and doxycycline [108–110].

Growth hormones and birth control hormones (Figure 2) have been mostly focused for sustained release formulation. Encapsulation of growth hormone in biodegradable PLGA microspheres has been a typical technique to prolong the effect of the drug. Human growth hormone, a somatotropic hormone to treat growth hormone deficiency (GHD), chronic renal insufficiency, Turner’ s syndrome, and cachexia secondary to AIDS, has been developed to reduce the need for frequent administrations by maintaining *in vivo* drug levels in the therapeutic range [28,111,112]. On the other hand, octreotide, a synthetic anti-somatotropic agent for the treatment of acromegaly and endocrine tumors, has been formulated in PLGA microspheres and commercialized as Sandostatin^®^ LAR^®^ depot (Novartis Pharma, Basel, Switzerland) on a monthly basis [113]. The use of polymers to deliver birth control hormones has evolved over the years. The first system, Norplant^®^, consisted of six levonorgestrel contraceptive implants for a five year duration of use. By replacing the initial model of silastic capsules containing steroid crystals with a solid mixture of the steroid and a polymer (rods) covered by a release-regulating silastic membrane, it was possible to release the same amount of contraceptive steroid delivered by six capsules through two rods, which is a second generation implant system, Jadelle^®^ [114]. However, these products are silicone based devices, which are non-biodegradable with considerable long-term toxicities. Consequently, the devices need to be removed after depletion of the drug. To overcome this problem, PLGA microspheres have been studied for implantation using levonorgestrel under the skin without special surgery [115–118].

Patient compliance rates are notoriously poor in antipsychotic medications due to the nature of the disease, troublesome side effects, and symptom recurrence. Undoubtedly, sustained and controlled release systems offer many advantages in the delivery of antipsychotics, reducing the frequency of dosing and enhancing drug bioavailability [119]. Haloperidol [120], risperidone [121,122], clozapine [123], and olanzapine [124] have been, and are being, studied for long acting particulate formulations.

There are oral dosage formulations for which osmotic pumping is the major release mechanism. In this system, osmotic pressure is used as the driving force to induce drug release in a predictable and uniform manner. The osmotic pump consists of a solid core containing drug, alone or with an osmotic agent, surrounded by a semi-permeable membrane, which has a delivery pore. When this device is placed in water, the water is imbibed osmotically into the core, thereby pushing a volume of saturated drug solution through the delivery orifice in a programmed manner [125,126]. Propranolol [127], nifedipine [128], allopurinol [129], ferulate [130], diclofenac [131], and pseudoephedrine [132] have been formulated as osmotic pump controlled release formulations.

## 5. Types of Polymeric Pharmaceutical/Drug Delivery Particles

### 5.1. Microparticles for Controlled Release Delivery

Due to the development of particulate drug delivery system, current formulations in the market for delivering proteins and peptides have reduced administration from once a month to every three months. Microparticles are particles between 0.1 and 100 μm in size. Kang and Singh studied the effect of additives on the physicochemical characteristics and *in vitro* release of a model protein, bovine serum albumin (BSA) [133]. The addition of hydrophobic tricaprin additives with low molecular weight PEG-100, results in further release of BSA from PLGA microspheres. The difference in the release profiles between control and additive containing microspheres is closely related to their surface morphology.

Blanco and Alonso compared the size effect of preparation method, w/o/w solvent extraction *vs.* o/o solvent evaporation, and encapsulation efficiency along with using stabilizer [134]. The size of microspheres prepared by two different methods depended on the intrinsic viscosity of the polymer solution. Microspheres using the w/o/w solvent extraction method showed a size increase, as intrinsic viscosity of the polymer solution increased, while the size of microsphere prepared by o/o solvent evaporation was increased with low viscosity polymer. Co-encapsulation of a stabilizer, poloxamer 188 or 331, induced lower loading efficiency and slower release of BSA. Without stabilizer, protein release is mainly influenced by polymer erosion rate and forming water-filled channels.

The effect of protein molecular weight (MW) on release kinetics from polymeric microspheres was studied using the phase inversion technique. The mechanism of release from microspheres appeared to be dependent on protein MW for microspheres with low loading (0.5–1.6%), whereas that is independent with high loadings (4.8–6.9%). At low loading, release of larger MW proteins was dependent on diffusion through pores for the duration of the study, while smaller MW proteins seemed to depend on diffusion through pores initially and on degradation at later times [135].

Tissue engineering in the context of controlled release drug delivery has been the subject of interesting recent research for drug delivery to bone tissue. Polymer microspheres as drug delivery carriers have been incorporated in 3D scaffolds for bone tissue controlled drug delivery [136–138]. Additionally, protein and small molecule therapeutics to promote bone growth have been incorporated in polymeric devices and in PLGA microspheres for controlled drug delivery to bone [139–143].

### 5.2. Nanoparticles for Controlled Release Delivery

The area of nanoparticle drug delivery is gaining much attention in recent years for a variety of administration routes, including pulmonary nanomedicine delivery [144]. To improve the bioavailability of PLGA nanoparticles, Barichello *et al.* formulated surface bound peptides using nanoprecipitation solvent displacement method [145]. Insulin was preferentially surface bound on the PLGA nanoparticles and the amount of insulin encapsulated into nanoparticles was related to composition and pH of the buffer solution; the optimal pH was close to the isoelectric point of insulin.

Insulin-loaded PLGA nanoparticles were prepared by w/o/w and s/o/w encapsulation methods with a stabilizer, Pluronic F68. Comparing the nanoparticles prepared by s/o/w method, the insulin release rate was higher for the batches prepared by w/o/w method containing stabilizers. Also the presence of stabilizers resulted in a sustained release of insulin, therefore a prolonged reduction of blood glucose level in diabetic rats [146].

Magnetically modulated nanoparticles are used for developing *in vivo* imaging and delivering drugs to targeted sites, such as tumors. Non-targeted applications of magnetic nanospheres include their use as contrast agents (MRI) and as drug carriers that can be activated by a magnet applied outside the body [147]. In another study, this magnetic force was used to improve the efficiency of orally delivered protein therapeutics. When the external magnetic field was applied to the intestine, the transit time of magnetic particles slowed down; therefore, the residence time of the orally delivered particles in small intestine is extended and absorption of protein increases [148].

## 6. Manufacturing/Particle Engineering Design of Polymeric Microparticles and Nanoparticles

### 6.1. Double-Emulsion Evaporation Methods

As a considerable number of hydrophobic drugs are soluble in various water-immiscible organic solvent and are poorly soluble in water. By emulsion/solvent evaporation technique, both drug and biodegradable polymer are first dissolved in a solvent, mostly methylene chloride. The resulting organic oil phase is emulsified in an aqueous phase making o/w emulsion. Volatile solvents can be removed from this emulsion by evaporation [7]. However, for drugs that do not show a high solubility in methylene chloride, it can be replaced with butyl acetate, ethyl acetate, ethyl formate, or methylene ketone [7]. Alternatively, a cosolvent may be added to methylene chloride. For hydrophilic peptides or proteins, they are either dispersed in an organic solution of polymer or preferably processed in an aqueous solution of water-in-oil (w/o) emulsion resulting in a w/o or a w/o/w emulsion system [149]. However, o/w or w/o/w methods are predicted to result in low encapsulation efficiencies due to a flux of drugs from the dispersed phase to the larger volume of the continuous phase during manufacturing process [7]. In addition, proteins encapsulated by w/o or w/o/w techniques into particles are susceptible to denaturation resulting in a loss of biological activity, aggregation, oxidation and cleavage, especially at the aqueous phase-solvent interface [149]. In order to improve protein integrity, the use of stabilizers and surfactants are suggested during the primary emulsion phase.

### 6.2. Supercritical Fluid (SCF) Technology

Substances become supercritical fluids (SCF) when placed above their critical point, which exhibit the flow properties of a gas and the dissolving behavior of a liquid. Their solvent power is affected by density, temperature and pressure. Many excellent reviews exist on this cutting-edge particle engineering design technique that has found increasing utility in novel delivery systems for many routes of administration, particularly in non-invasive pulmonary delivery via pharmaceutical inhalation aerosols [150–153].

There are two possible processes for the drug and matrix polymer to be either dissolved or melted in the SCF and afterwards form particles following either the rapid expansion from supercritical solution (RESS) or from gas-saturated solution (PGSS) process. The RESS process, fine particles formed using the supercritical fluid as a good solvent, has two steps: (1) dissolving the solute into a supercritical fluid; and (2) formation of the solute as a microparticle due to rapid supersaturation [154]. CO_2_ is an attractive solvent for a variety of chemical and industrial processes, since it is abundant, inexpensive, non-toxic, and a relatively accessible critical point, *i.e.*, T_c_ = 304.2 K and P_c_ = 7.37 MPa [154–156]. In the PGSS process, the supercritical fluid or dense gas is used as a solute. Polar or high molecular weight substances, such as proteins, are difficult to dissolve in CO_2_, which has no polarity. However, the ability of CO_2_ to diffuse into organic compounds enables the formation of composite particles in the PGSS process. The organic compounds will mainly constitute polymers and CO_2_ lowers the melting point and decreases the viscosity of a compound with an increase in its concentration. As a result, the compounds are melted in a compressed gas and the concentration of a gas in a molten solute increases with pressure forming a saturated solution. When this solution is rapidly depressurized through a nozzle, composite microcapsules can be formed due to the release of gas from the condensed phase [154].

### 6.3. Supercritical Antisolvent Method

CO_2_ is the most common supercritical fluid used in pharmaceutical applications due to its relatively accessible critical point, abundance, and minimal toxicity [150,155]. In addition to RESS and PGSS, the antisolvent method utilizes CO_2_ as an antisolvent for particle fabrication. Antisolvent methods have the advantage of utilizing the high miscibility of supercritical fluids with organic solvents which have high dissolving power for the compound [155]. The techniques include the supercritical antisolvent (GAS/SAS), the precipitation with compressed supercritical fluid (PCA), aerosol solvent extraction system (ASES) and the solution-enhanced dispersion by supercritical fluids (SEDS) processes. The principle of the supercritical antisolvent method (GAS/SAS) is based on a rapid decrease in the solubilization power of a solvent by addition of a second fluid as antisolvent. Adding the antisolvent expands the organic solution thereby dissolving the solute inducing supersaturation of the solution. The precipitated particles are washed with the antisolvent to remove remaining solvent [154]. Particle size can be regulated by several factors, such as temperature, pressure and composition [154]. In contrast to the one-way mass transfer of the CO_2_ into the organic phase in the GAS process, in the PCA process a two-way mass transfer occurs. The organic solvent diffuses into the CO_2_, and the CO_2_ diffuses into the organic phase. In the ASES process, the drug and polymer are dissolved or dispersed in an organic solvent, *i.e.*, generally soluble in the supercritical CO_2_, that is sprayed into a supercritical CO_2,_ then extracted, resulting in the formation of solid microparticles [157,158]. In the SEDS process, the particle formation is attributed to the mass transfer of the supercritical fluid into the sprayed droplet and to the rate of solvent transfer into the supercritical phase. Notably, a high mass transfer leads to a smaller particle size distribution with less agglomeration [159].

### 6.4. Spray Drying Particle Engineering Design

Spray drying has been widely used in the efficient design and production of food and pharmaceutical particles, especially particles designed for use in pharmaceutical inhalation aerosols [151,160]. Spray drying [151] comprises of four steps: (1) atomization of the feed solution into fine droplets in a spray; (2) spray-air contact involving intimate flow and mixing; (3) drying of sprayed droplets at elevated temperatures; and (4) separation of dried particles from the air [160]. In order to control the various particle characteristics, the operating parameters of the spray drying process such as atomization pressure, feed rate, airflow, inlet temperature, outlet temperature, and the size of nozzle orifice all must be controlled [161]. Generally, a smaller nozzle orifice, faster atomization airflow, and a low feed concentration generate a larger particle size [162,163]. To modify the particle morphology, the feed solvent type [164] or optimizing the outlet drying temperature can be done [165]. By adding Tween 20 and lactose to the feed solution, the particles with rougher surfaces can be obtained [165].

Spray-freeze drying is based on the atomization of an aqueous drug solution via a two-fluid or an ultrasonic nozzle into a spray chamber which is filled with a cryogenic liquid, *i.e.*, liquid nitrogen, or halocarbon refrigerant, e.g., chlorofluorocarbon, hydrofluorocarbon [166]. Once the liquid droplets contact the cryogenic medium, it solidifies quickly due to the high heat-transfer rate. After the spraying process is completed, the collected contents are lyophilized and frozen solvent is removed by vacuum or atmospheric freeze-drying [167]. To obtain a smaller particle size, the mass flow ratio of atomized nitrogen to liquid feed, which has the most significant influence to particle size, should be increased [168]. Spray freeze-drying can be exploited to create small microparticles and nanoparticles [169,170].

## 7. Marketed Controlled Release Polymeric Pharmaceutical Products and Clinical Trials

Administration of a variety of drugs encapsulated in polymeric particles has been extensively investigated leading to complete absorption of drugs in systemic circulation and control drug release over a predetermined time span in days to weeks to months, resulting in increased patient compliance and maximal therapeutic effects. Lupron^®^ Depot is a microsphere formulation of leuprolide with duration of one, three or four months using PLA or PLGA in the treatment of prostate cancer and endometriosis. Nutropin^®^, a commercial PLGA microsphere formulation product of human growth hormone, is used for two weeks or one month duration. As a synthetic anti-somatotropic agent for the treatment of acromegaly and endocrine tumors, Octreotide encapsulated in PLGA microspheres, commercialized as Sandostatin^®^ LAR^®^ is taken on a monthly basis. In addition, Trelstar^®^ Depot for triptorelin, Suprecur MP^®^ for buserelin, Somatuline LA^®^ for lanreotide, Arestin^®^ for minocycline, Risperdal Consta^®^ for risperidone have been commercialized as a parenteral microsphere formulation products for extended duration [171–176]. Micellar nanoparticles incorporating paclitaxel or cisplatin are in their clinical trials [177]. There are also oral dosage formulation commercial products for which osmotic pressure is the major driving force in release mechanism, including Procardia XL^®^ for nifedipine (Figure 2) and Glucotrl XL^®^ for glipizide [47,48,178].

## 8. Conclusions

Biodegradable and biocompatible materials for pharmaceutical dosage forms have enabled the advancement of pharmaceuticals by providing better therapy and disease state management for patients through controlled release. Controlled release delivery is available for many routes of administration and offers many advantages over immediate release delivery. These advantages include reduced dosing frequency, better therapeutic control, fewer side effects, and, consequently, these dosage forms are well accepted by patients. Advancements in polymer material science, particle engineering design, manufacture, and nanotechnology have led the way to the introduction of several marketed controlled release products containing polypeptide drugs and protein drugs that retain their therapeutic activity over pharmaceutical timescales following encapsulation in biodegradable materials.

## Figures and Tables

**Table 1:** Polymeric inactive ingredients for FDA-approved drug products.

	Polymeric inactive ingredients for FDA-approved drug products
**Polyester-based synthetic polymers**	PLGA (for IM, SC uses) Poloxamer (for oral, topical, IV opthalmic, SC uses) Polyvinylpyrrolidone ethylcellulose (for oral use) Sodium pyrrolidone carboxylate (for topical use) Povidone (for oral, intra-articular, IM, Intrauterine, topical, SC, respiratory, opthalmic uses) PLA (for IM use) PEG (for oral, respiratory, topical, IM, IV, opthalmic uses) PVA (for auricular, IM, intraocular, topical uses) KOLLIDON VA 64 (for oral use)
**Natural-origin polymers**	Starch (for oral, IV, IM, topical) Hyaluronate (for intra-articular, IM, intravitreal, topical uses), Human albumin (for IV, SC, Oral uses) Gelatin (for IM, SC, IV, oral topical uses) Alginic acid (for opthalmic and oral uses) Collagen (for topical use)

## References

[1] Minko T Sinko PJ Drug Delivery Systems-Controlled Drug Release Martns Physical Pharmacy and Pharmaceutical Sciences Lippincott Williams & Wilkins Baltimore, MD, USA 2006 667 672

[2] Jantzen GM Robinson JR Banker GS Rhodes CT Sustained- and Controlled-Release Drug Delivery Systems Modern Pharmaceutics Marcel Dekker, Inc New York, NY, USA 2002 501 528

[3] Longer MA Robinson JR Gennaro AR Sustained-Release Drug Delivery Systems Remington’s Pharmaceutical Sciences Mack Publishing Company Easton, PA, USA 1990 1676 1693

[4] Ebube NK Hikal AH Wyandt CM Beer DC Miller LG Jones AB Sustained release of acetaminophen from heterogeneous matrix tablets: Influence of polymer ratio, polymer loading, and co-active on drug release Pharm. Dev. Technol 1997 2 161 170 9552442 10.3109/10837459709022621

[5] Miyazaki Y Yakou S Takayama K Study on jelly fig extract as a potential hydrophilic matrix for controlled drug delivery Int. J. Pharm 2004 287 39 46 15541910 10.1016/j.ijpharm.2004.08.023

[6] Panyam J Dali MM Sahoo SK Ma W Chakravarthi SS Amidon GL Levy RJ Labhasetwar V Polymer degradation and *in vitro* release of a model protein from poly (D,L-lactide-co-glycolide) nano- and microparticles J. Control. Release 2003 92 173 187 14499195 10.1016/s0168-3659(03)00328-6

[7] Wischke C Schwendeman SP Principles of encapsulating hydrophobic drugs in PLA/PLGA microparticles Int. J. Pharm 2008 364 298 327 18621492 10.1016/j.ijpharm.2008.04.042

[8] Fundueanu G Constantin M Dalpiaz A Bortolotti F Cortesi R Ascenzi P Menegatti E Preparation and characterization of starch/cyclodextrin bioadhesive microspheres as platform for nasal administration of Gabexate Mesylate (Foy) in allergic rhinitis treatment Biomaterials 2004 25 159 170 14580919 10.1016/s0142-9612(03)00477-0

[9] Peng H Xiao Y Mao X Chen L Crawford R Whittaker AK Amphiphilic triblock copolymers of methoxy-poly(ethylene glycol)-b-poly(L-lactide)-b-poly(L-lysine) for enhancement of osteoblast attachment and growth Biomacromolecules 2009 10 95 104 19063715 10.1021/bm800937g

[10] Na DH DeLuca PP PEGylation of octreotide: I. Separation of positional isomers and stability against acylation by poly (D,L-lactide-co-glycolide) Pharm. Res 2005 22 736 742 15906168 10.1007/s11095-005-2589-4

[11] Na DH Lee JE Jang SW Lee KC Formation of acylated growth hormone-releasing peptide-6 by poly(lactide-co-glycolide) and its biological activity AAPS Pharm. Sci. Tech 2007 8 43 10.1208/pt0802043 PMC2750358 17622118

[12] Berkland C King M Cox A Kim K Pack DW Precise control of PLG microsphere size provides enhanced control of drug release rate J. Control. Release 2002 82 137 147 12106984 10.1016/s0168-3659(02)00136-0 PMC4140625

[13] Quaglia F Ostacolo L Nese G De Rosa G La Rotonda MI Palumbo R Maglio G Microspheres made of poly (epsilon-caprolactone)-based amphiphilic copolymers: potential in sustained delivery of proteins Macromol. Biosci 2005 5 945 954 16208680 10.1002/mabi.200500108

[14] Wei H Zhang XZ Zhou Y Cheng SX Zhuo RX Self-assembled thermoresponsive micelles of poly (N-isopropylacrylamide-b-methyl methacrylate) Biomaterials 2006 27 2028 2034 16225918 10.1016/j.biomaterials.2005.09.028

[15] Kumar N Ravikumar MN Domb AJ Biodegradable block copolymers Adv. Drug Deliv. Rev 2001 53 23 44 11733116 10.1016/s0169-409x(01)00219-8

[16] Choi S Baudys M Kim SW Control of blood glucose by novel GLP-1 delivery using biodegradable triblock copolymer of PLGA-PEG-PLGA in type 2 diabetic rats Pharm. Res 2004 21 827 831 15180341 10.1023/b:pham.0000026435.27086.94

[17] Jaraswekin S Prakongpan S Bodmeier R Effect of poly(lactide-co-glycolide) molecular weight on the release of dexamethasone sodium phosphate from microparticles J. Microencapsul 2007 24 117 128 17454423 10.1080/02652040701233655

[18] Wei G Pettway GJ McCauley LK Ma PX The release profiles and bioactivity of parathyroid hormone from poly(lactic-co-glycolic acid) microspheres Biomaterials 2004 25 345 352 14585722 10.1016/s0142-9612(03)00528-3

[19] Tracy MA Ward KL Firouzabadian L Wang Y Dong N Qian R Zhang Y Factors affecting the degradation rate of poly(lactide-co-glycolide) microspheres *in vivo* and *in vitro* Biomaterials 1999 20 1057 1062 10378806 10.1016/s0142-9612(99)00002-2

[20] Tsukada Y Hara K Bando Y Huang CC Kousaka Y Kawashima Y Morishita R Tsujimoto H Particle size control of poly(dl-lactide-co-glycolide) nanospheres for sterile applications Int. J. Pharm 2009 370 196 201 19100320 10.1016/j.ijpharm.2008.11.019

[21] Sinha VR Bansal K Kaushik R Kumria R Trehan A Poly-epsilon-caprolactone microspheres and nanospheres: An overview Int. J. Pharm 2004 278 1 23 15158945 10.1016/j.ijpharm.2004.01.044

[22] Abuchowski A McCoy JR Palczuk NC van Es T Davis FF Effect of covalent attachment of polyethylene glycol on immunogenicity and circulating life of bovine liver catalase J. Biol. Chem 1977 252 3582 3586 16907

[23] Esposito P Barbero L Caccia P Caliceti P D’Antonio M Piquet G Veronese FM PEGylation of growth hormone-releasing hormone (GRF) analogues Adv. Drug Deliv. Rev 2003 55 1279 1291 14499707 10.1016/s0169-409x(03)00109-1

[24] Wu Z Chen H Liu X Zhang Y Li D Huang H Protein Adsorption on Poly(N-vinylpyrrolidone)-Modified Silicon Surfaces Prepared by Surface-Initiated Atom Transfer Radical Polymerization Langmuir 2009 25 2900 2906 19437703 10.1021/la8037523

[25] Mawad D Poole-Warren LA Martens P Koole LH Slots TL van Hooy-Corstjens CS Synthesis and characterization of radiopaque iodine-containing degradable PVA hydrogels Biomacromolecules 2008 9 263 268 18047286 10.1021/bm700754m

[26] Fernandes PA Tzvetkov G Fink RH Paradossi G Fery A Quantitative analysis of scanning transmission X-ray microscopy images of gas-filled PVA-based microballoons Langmuir 2008 24 13677 13682 18980347 10.1021/la801898t

[27] D’Souza SS DeLuca PP Methods to assess *in vitro* drug release from injectable polymeric particulate systems Pharm. Res 2006 23 460 474 16400516 10.1007/s11095-005-9397-8

[28] Park EJ Na DH Lee KC *In vitro* release study of mono-PEGylated growth hormone-releasing peptide-6 from PLGA microspheres Int. J. Pharm 2007 343 281 283 17644286 10.1016/j.ijpharm.2007.06.005

[29] Sehra S Dhake AS Formulation and evaluation of sustained release microspheres of poly-lactide-co-glycolide containing tamoxifen citrate J. Microencapsul 2005 22 521 528 16361195 10.1080/02652040500162170

[30] Dailey LA Wittmar M Kissel T The role of branched polyesters and their modifications in the development of modern drug delivery vehicles J. Control. Release 2005 101 137 149 15588900 10.1016/j.jconrel.2004.09.003

[31] Muschert S Siepmann F Leclercq B Carlin B Siepmann J Drug release mechanisms from ethylcellulose: PVA-PEG graft copolymer-coated pellets Eur. J. Pharm. Biopharm 2009 72 130 137 19146955 10.1016/j.ejpb.2008.12.007

[32] Mumper RJ Duguid JG Anwer K Barron MK Nitta H Rolland AP Polyvinyl derivatives as novel interactive polymers for controlled gene delivery to muscle Pharm. Res 1996 13 701 709 8860424 10.1023/a:1016039330870

[33] Hayama M Yamamoto K Kohori F Uesaka T Ueno Y Sugaya H Itagaki I Sakai K Nanoscopic behavior of polyvinylpyrrolidone particles on polysulfone/polyvinylpyrrolidone film Biomaterials 2004 25 1019 1028 14615167 10.1016/s0142-9612(03)00629-x

[34] Buhler V Kollidon^®^ — Polyvinylpyrrolidone for the Pharmaceutical Industry BASF LUD, Germany 1999

[35] Sahoo J Murthy PN Biswal SM Formulation of sustained-release dosage form of verapamil hydrochloride by solid dispersion technique using Eudragit RLPO or Kollidon SR AAPS Pharm. Sci. Technol 2009 10 27 33 10.1208/s12249-008-9175-0 PMC2663666 19145487

[36] Reis RL Neves NM Mano JF Gomes ME Marques AP Azevedo HS Natural-Based Polymers for Biomedical Applications CRC Press-Woodhead Publishing Limited Boca Raton, FL, USA 2008

[37] Malafaya PB Silva GA Reis RL Natural-origin polymers as carriers and scaffolds for biomolecules and cell delivery in tissue engineering applications Adv. Drug Deliv. Rev 2007 59 207 233 17482309 10.1016/j.addr.2007.03.012

[38] Bonacucina G Di Martino P Piombetti M Colombo A Roversi F Palmieri GF Effect of plasticizers on properties of pregelatinised starch acetate (Amprac 01) free films Int. J. Pharm 2006 313 72 77 16540269 10.1016/j.ijpharm.2006.01.046

[39] Ma X Jian R Chang PR Yu J Fabrication and characterization of citric acid-modified starch nanoparticles/plasticized-starch composites Biomacromolecules 2008 9 3314 3320 18844405 10.1021/bm800987c

[40] Coucke D Schotsaert M Libert C Pringels E Vervaet C Foreman P Saelens X Remon JP Spray-dried powders of starch and crosslinked poly(acrylic acid) as carriers for nasal delivery of inactivated influenza vaccine Vaccine 2009 27 1279 1286 19114075 10.1016/j.vaccine.2008.12.013

[41] Wei L Cai C Lin J Chen T Dual-drug delivery system based on hydrogel/micelle composites Biomaterials 2009 30 2606 2613 19162320 10.1016/j.biomaterials.2009.01.006

[42] Ozbas-Turan S Akbuga J Aral C Controlled release of interleukin-2 from chitosan microspheres J. Pharm. Sci 2002 91 1245 1251 11977100 10.1002/jps.10122

[43] d’Ayala GG Malinconico M Laurienzo P Marine derived polysaccharides for biomedical applications: chemical modification approaches Molecules 2008 13 2069 2106 18830142 10.3390/molecules13092069 PMC6245343

[44] Pajic-Lijakovic I Plavsic M Nedovic V Bugarski B Investigation of Ca-alginate hydrogel rheological behaviour in conjunction with immobilized yeast cell growth dynamics J. Microencapsul 2007 24 420 429 17578732 10.1080/02652040701362843

[45] Liao YH Jones SA Forbes B Martin GP Brown MB Hyaluronan: pharmaceutical characterization and drug delivery Drug Delivery 2005 12 327 342 16253949 10.1080/10717540590952555

[46] Kang JY Chung CW Sung JH Park BS Choi JY Lee SJ Choi BC Shim CK Chung SJ Kim DD Novel porous matrix of hyaluronic acid for the three-dimensional culture of chondrocytes Int. J. Pharm 2009 369 114 120 19059468 10.1016/j.ijpharm.2008.11.008

[47] Lin W Coombes AG Davies MC Davis SS Illum L Preparation of sub-100 nm human serum albumin nanospheres using a pH-coacervation method J. Drug. Target 1993 1 237 243 8069565 10.3109/10611869308996081

[48] Weber C Coester C Kreuter J Langer K Desolvation process and surface characterisation of protein nanoparticles Int. J. Pharm 2000 194 91 102 10601688 10.1016/s0378-5173(99)00370-1

[49] Wang G Siggers K Zhang S Jiang H Xu Z Zernicke RF Matyas J Uludag H Preparation of BMP-2 containing bovine serum albumin (BSA) nanoparticles stabilized by polymer coating Pharm. Res 2008 25 2896 2909 18709447 10.1007/s11095-008-9692-2

[50] Friess W Collagen-biomaterial for drug delivery Eur. J. Pharm. Biopharm 1998 45 113 136 9704909 10.1016/s0939-6411(98)00017-4

[51] Schoof H Apel J Heschel I Rau G Control of pore structure and size in freeze-dried collagen sponges J. Biomed. Mater. Res 2001 58 352 357 11410892 10.1002/jbm.1028

[52] Sun XD Jeng L Bolliet C Olsen BR Spector M Non-viral endostatin plasmid transfection of mesenchymal stem cells via collagen scaffolds Biomaterials 2009 30 1222 1231 19059640 10.1016/j.biomaterials.2008.10.020

[53] Young S Wong M Tabata Y Mikos AG Gelatin as a delivery vehicle for the controlled release of bioactive molecules J. Control. Release 2005 109 256 274 16266768 10.1016/j.jconrel.2005.09.023

[54] Adams ML Lavasanifar A Kwon GS Amphiphilic block copolymers for drug delivery J. Pharm. Sci 2003 92 1343 1355 12820139 10.1002/jps.10397

[55] Kataoka K Harada A Nagasaki Y Block copolymer micelles for drug delivery: design, characterization and biological significance Adv. Drug Deliv. Rev 2001 47 113 131 11251249 10.1016/s0169-409x(00)00124-1

[56] Otsuka H Nagasaki Y Kataoka K PEGylated nanoparticles for biological and pharmaceutical applications Adv. Drug Deliv. Rev 2003 55 403 419 12628324 10.1016/s0169-409x(02)00226-0

[57] Lee PY Li Z Huang L Thermosensitive hydrogel as a Tgf-beta1 gene delivery vehicle enhances diabetic wound healing Pharm. Res 2003 20 1995 2000 14725365 10.1023/b:pham.0000008048.58777.da

[58] Frauke PK Breitenbach A Zange-Volland R Kissel T Brush-like branched biodegradable polyesters, part III. Protein release from microspheres of poly(vinyl alcohol)-graft-poly(D,L-lactic- co-glycolic acid) J. Control. Release 2001 73 7 20 11337055 10.1016/s0168-3659(01)00231-0

[59] Jung T Breitenbach A Kissel T Sulfobutylated poly(vinyl alcohol)-graft-poly(lactide-co-glycolide) s facilitate the preparation of small negatively charged biodegradable nanospheres J. Control. Release 2000 67 157 169 10825550 10.1016/s0168-3659(00)00201-7

[60] Jung T Kamm W Breitenbach A Hungerer KD Hundt E Kissel T Tetanus toxoid loaded nanoparticles from sulfobutylated poly(vinyl alcohol)-graft-poly(lactide-co-glycolide): evaluation of antibody response after oral and nasal application in mice Pharm. Res 2001 18 352 360 11442276 10.1023/a:1011063232257

[61] Jung T Kamm W Breitenbach A Klebe G Kissel T Loading of tetanus toxoid to biodegradable nanoparticles from branched poly(sulfobutyl-polyvinyl alcohol)-g-(lactide-co-glycolide) nanoparticles by protein adsorption: a mechanistic study Pharm. Res 2002 19 1105 1113 12240935 10.1023/a:1019833822997

[62] Batrakova EV Kabanov AV Pluronic block copolymers: evolution of drug delivery concept from inert nanocarriers to biological response modifiers J. Control. Release 2008 130 98 106 18534704 10.1016/j.jconrel.2008.04.013 PMC2678942

[63] Kabanov AV Batrakova EV Alakhov VY Pluronic block copolymers for overcoming drug resistance in cancer Adv. Drug Deliv. Rev 2002 54 759 779 12204601 10.1016/s0169-409x(02)00047-9

[64] Kabanov AV Batrakova EV Alakhov VY Pluronic block copolymers as novel polymer therapeutics for drug and gene delivery J. Control. Release 2002 82 189 212 12175737 10.1016/s0168-3659(02)00009-3

[65] Dhanikula AB Panchagnula R Localized paclitaxel delivery Int. J. Pharm 1999 183 85 100 10361159 10.1016/s0378-5173(99)00087-3

[66] Wang Y Yu L Han L Sha X Fang X Difunctional Pluronic copolymer micelles for paclitaxel delivery: synergistic effect of folate-mediated targeting and Pluronic-mediated overcoming multidrug resistance in tumor cell lines Int. J. Pharm 2007 337 63 73 17289311 10.1016/j.ijpharm.2006.12.033

[67] Huh KM Lee SC Cho YW Lee J Jeong JH Park K Hydrotropic polymer micelle system for delivery of paclitaxel J. Control. Release 2005 101 59 68 15588894 10.1016/j.jconrel.2004.07.003

[68] Cheon LS Kim C Chan KI Chung H Young JS Polymeric micelles of poly(2-ethyl-2- oxazoline)-block-poly(epsilon-caprolactone) copolymer as a carrier for paclitaxel J. Control. Release 2003 89 437 446 12737846 10.1016/s0168-3659(03)00162-7

[69] Le GD Gori S Luo L Lessard D Smith DC Yessine MA Ranger M Leroux JC Poly(N-vinylpyrrolidone)-block-poly(D,L-lactide) as a new polymeric solubilizer for hydrophobic anticancer drugs: *in vitro* and *in vivo* evaluation J. Control. Release 2004 99 83 101 15342183 10.1016/j.jconrel.2004.06.018

[70] Xiong XB Uludag H Lavasanifar A Biodegradable amphiphilic poly(ethylene oxide)-block-polyesters with grafted polyamines as supramolecular nanocarriers for efficient siRNA delivery Biomaterials 2009 30 242 253 18838158 10.1016/j.biomaterials.2008.09.025

[71] Sun TM Du JZ Yan LF Mao HQ Wang J Self-assembled biodegradable micellar nanoparticles of amphiphilic and cationic block copolymer for siRNA delivery Biomaterials 2008 29 4348 4355 18715636 10.1016/j.biomaterials.2008.07.036

[72] Gupte A Ciftci K Formulation and characterization of Paclitaxel, 5-FU and Paclitaxel +5-FU microspheres Int. J. Pharm 2004 276 93 106 15113618 10.1016/j.ijpharm.2004.02.023

[73] Roullin VG Mege M Lemaire L Cueyssac JP Venier-Julienne MC Menei P Gamelin E Benoit JP Influence of 5-fluorouracil-loaded microsphere formulation on efficient rat glioma radiosensitization Pharm. Res 2004 21 1558 1563 15497679 10.1023/b:pham.0000041448.22771.48

[74] Shuai X Ai H Nasongkla N Kim S Gao J Micellar carriers based on block copolymers of poly(epsilon-caprolactone) and poly(ethylene glycol) for doxorubicin delivery J. Control. Release 2004 98 415 426 15312997 10.1016/j.jconrel.2004.06.003

[75] Yadav AK Mishra P Mishra AK Jain S Agrawal GP Development and characterization of hyaluronic acid-anchored PLGA nanoparticulate carriers of doxorubicin Nanomedicine 2007 3 246 257 18068091 10.1016/j.nano.2007.09.004

[76] Bae Y Nishiyama N Kataoka K *In vivo* antitumor activity of the folate-conjugated pH-sensitive polymeric micelle selectively releasing adriamycin in the intracellular acidic compartments Bioconjug. Chem 2007 18 1131 1139 17488066 10.1021/bc060401p

[77] Maeda M Moriuchi S Sano A Yoshimine T New drug delivery system for water-soluble drugs using silicone and its usefulness for local treatment: application of GCV-silicone to GCV/HSV-tk gene therapy for brain tumor J. Control. Release 2002 84 15 25 12399164 10.1016/s0168-3659(02)00236-5

[78] Park K Yang JH Choi Y Lee C Kim SY Byun Y Chemoprevention of 4-NQO-induced oral carcinogenesis by co-administration of all-trans retinoic acid loaded microspheres and celecoxib J. Control. Release 2005 104 167 179 15866343 10.1016/j.jconrel.2005.01.013

[79] Sinha VR Bhinge JR Kumria R Kumar M Development of pulsatile systems for targeted drug delivery of celecoxib for prophylaxis of colorectal cancer Drug Delivery 2006 13 221 225 16556575 10.1080/10717540500309180

[80] D’Souza R Mutalik S Udupa N *In Vitro* and *in Vivo* preparation evaluations of bleomycin implants and microspheres Prepared with DL-poly (lactide-co-glycolide) Drug Dev. Ind. Pharm 2006 32 175 184 16537198 10.1080/03639040500466064

[81] Shenoy DB D’Souza RJ Udupa N Poly(DL-lactide-co-glycolide) microporous microsphere-based depot formulation of a peptide-like antineoplastic agent J. Microencapsul 2002 19 523 535 12396388 10.1080/02652040210141084

[82] Chen J Cynkowski T Guo H Qin K Cabral-Lilly D Walters K Ashton P Morphine pharmacokinetics following intra-articular administration of a novel sustained release opioid (CDS-PM-101) for the relief of post-operative orthopaedic pain J. Control. Release 2005 101 359 360 15822207

[83] Morales ME Gallardo LV Calpena AC Domenech J Ruiz MA Comparative study of morphine diffusion from sustained release polymeric suspensions J. Control. Release 2004 95 75 81 15013234 10.1016/j.jconrel.2003.11.002

[84] Liu FI Kuo JH Sung KC Hu OY Biodegradable polymeric microspheres for nalbuphine prodrug controlled delivery: *in vitro* characterization and *in vivo* pharmacokinetic studies Int. J. Pharm 2003 257 23 31 12711158 10.1016/s0378-5173(03)00110-8

[85] Tiwari SB Murthy TK Pai MR Mehta PR Chowdary PB Controlled release formulation of tramadol hydrochloride using hydrophilic and hydrophobic matrix system AAPS Pharm. Sci. Technol 2003 4 E31 10.1208/pt040331 PMC2750624 14621963

[86] Dinarvand R Alimorad MM Amanlou M Akbari H *In vitro* release of clomipramine HCl and buprenorphine HCl from poly adipic anhydride (PAA) and poly trimethylene carbonate (PTMC) blends J. Biomed. Mater. Res. Part A 2005 75 185 191 10.1002/jbm.a.30398 16044413

[87] Kleppner SR Patel R McDonough J Costantini LC *In vitro* and *in vivo* characterization of a buprenorphine delivery system J. Pharm. Pharmacol 2006 58 295 302 16536895 10.1211/jpp.58.3.0002

[88] Seo SA Choi HS Khang G Rhee JM Lee HB A local delivery system for fentanyl based on biodegradable poly(L-lactide-co-glycolide) oligomer Int. J. Pharm 2002 239 93 101 12052694 10.1016/s0378-5173(02)00074-1

[89] Seo SA Khang G Rhee JM Kim J Lee HB Study on *in vitro* release patterns of fentanyl-loaded PLGA microspheres J. Microencapsulaytion 2003 20 569 579 10.1080/0265204031000148013 12909542

[90] Sendil D Bonney IM Carr DB Lipkowski AW Wise DL Hasirci V Antinociceptive effects of hydromorphone, bupivacaine and biphalin released from PLGA polymer after intrathecal implantation in rats Biomaterials 2003 24 1969 1976 12615487 10.1016/s0142-9612(02)00567-7

[91] Lu Y Zhang G Sun D Zhong Y Preparation and evaluation of biodegradable flubiprofen gelatin micro-spheres for intra-articular administration J. Microencapsul 2007 24 515 524 17654172 10.1080/02652040701433479

[92] Fernandez-Carballido A Herrero-Vanrell R Molina-Martinez IT Pastoriza P Sterilized ibuprofen-loaded poly(D,L-lactide-co-glycolide) microspheres for intra-articular administration: effect of gamma-irradiation and storage J. Microencapsul 2004 21 653 665 15762322 10.1080/09687860400008437

[93] Thakkar H Sharma RK Mishra AK Chuttani K Murthy RS Celecoxib incorporated chitosan microspheres: *in vitro* and *in vivo* evaluation J. Drug Targeting 2004 12 549 557 10.1080/10611860400010630 15621680

[94] Thakkar H Sharma RK Mishra AK Chuttani K Murthy RS Efficacy of chitosan microspheres for controlled intra-articular delivery of celecoxib in inflamed joints J. Pharm. Pharmacol 2004 56 1091 1099 15324477 10.1211/0022357044166

[95] Tuncay M Calis S Kas HS Ercan MT Peksoy I Hincal AA *In vitro* and *in vivo* evaluation of diclofenac sodium loaded albumin microspheres J. Microencapsul 2000 17 145 155 10738690 10.1080/026520400288382

[96] Tuncay M Calis S Kas HS Ercan MT Peksoy I Hincal AA Diclofenac sodium incorporated PLGA (50:50) microspheres: formulation considerations and *in vitro*/*in vivo* evaluation Int. J. Pharm 2000 195 179 188 10675695 10.1016/s0378-5173(99)00394-4

[97] La SB Okano T Kataoka K Preparation and characterization of the micelle-forming polymeric drug indomethacin-incorporated poly(ethylene oxide)-poly(beta-benzyl L-aspartate) block copolymer micelles J. Pharm. Sci 1996 85 85 90 8926590 10.1021/js950204r

[98] Shin IG Kim SY Lee YM Cho CS Sung YK Methoxy poly(ethylene glycol)/epsilon-caprolactone amphiphilic block copolymeric micelle containing indomethacin. I. Preparation and characterization J. Control. Release 1998 51 1 11 9685899 10.1016/s0168-3659(97)00164-8

[99] Sendil-Keskin D Altunay H Wise DL Hasirci V *In vivo* pain relief effectiveness of an analgesic-anesthetic carrying biodegradable controlled release rod systems J. Biomater. Sci., Polym. Ed 2003 14 497 514 12901434 10.1163/15685620360674218

[100] Loughlin RG Tunney MM Donnelly RF Murphy DJ Jenkins M McCarron PA Modulation of gel formation and drug-release characteristics of lidocaine-loaded poly(vinyl alcohol)-tetraborate hydrogel systems using scavenger polyol sugars Eur. J. Pharm. Biopharm 2008 69 1135 1146 18417328 10.1016/j.ejpb.2008.01.033

[101] Moss GP Gullick DR Woolfson AD McCafferty DF Mechanical characterization and drug permeation properties of tetracaine-loaded bioadhesive films for percutaneous local anesthesia Drug Dev. Ind. Pharm 2006 32 163 174 16537197 10.1080/03639040500466049

[102] Ratajczak-Enselme M Estebe JP Dollo G Chevanne F Bec D Malinovsky JM Ecoffey C Le Corre P Epidural, intrathecal and plasma pharmacokinetic study of epidural ropivacaine in PLGA-microspheres in sheep model Eur. J. Pharm. Biopharm 2009 72 54 61 19061956 10.1016/j.ejpb.2008.11.003

[103] Kiremitci AS Ciftci A Ozalp M Gumusderelioglu M Novel chlorhexidine releasing system developed from thermosensitive vinyl ether-based hydrogels J. Biomed. Mater. Res. Part B 2007 83 609 614 10.1002/jbm.b.30834 17471518

[104] Bigucci F Luppi B Musenga A Zecchi V Cerchiara T Chitosan salts coated with stearic acid as colon-specific delivery systems for vancomycin Drug Delivery 2008 15 289 293 18763159 10.1080/10717540802006468

[105] Nahar M Mishra D Dubey V Jain NK Development, characterization, and toxicity evaluation of amphotericin B-loaded gelatin nanoparticles Nanomedicine 2008 4 252 261 18502187 10.1016/j.nano.2008.03.007

[106] Jia Y Joly H Omri A Liposomes as a carrier for gentamicin delivery: development and evaluation of the physicochemical properties Int. J. Pharm 2008 359 254 263 18485632 10.1016/j.ijpharm.2008.03.035

[107] Lecaroz C Gamazo C Renedo MJ Blanco-Prieto MJ Biodegradable micro- and nanoparticles as long-term delivery vehicles for gentamicin J. Microencapsul 2006 23 782 792 17123922 10.1080/02652040600946886

[108] Gillissen M Steendam R van der Laan A Tijsma E Development of doxycycline-eluting delivery systems based on SynBiosys biodegradable multi-block copolymers J. Control. Release 2006 116 90 92 17718990 10.1016/j.jconrel.2006.09.066

[109] Mundargi RC Srirangarajan S Agnihotri SA Patil SA Ravindra S Setty SB Aminabhavi TM Development and evaluation of novel biodegradable microspheres based on poly(d,l-lactide-co-glycolide) and poly(epsilon-caprolactone) for controlled delivery of doxycycline in the treatment of human periodontal pocket: *in vitro* and *in vivo* studies J. Control. Release 2007 119 59 68 17331611 10.1016/j.jconrel.2007.01.008

[110] Giovagnoli S Tsai T DeLuca PP Formulation and release behavior of doxycycline-alginate hydrogel microparticles embedded into pluronic F127 thermogels as a potential new vehicle for doxycycline intradermal sustained delivery AAPS Pharm-Sci-Tech 2010 11 212 220 10.1208/s12249-009-9361-8 PMC2850495 20127210

[111] Capan Y Jiang G Giovagnoli S Na KH DeLuca PP Preparation and characterization of poly(D,L-lactide-co-glycolide) microspheres for controlled release of human growth hormone AAPS Pharm. Sci. Technol 2003 4 28 10.1208/pt040228 PMC2750591 12916910

[112] Chen S Singh J Controlled release of growth hormone from thermosensitive triblock copolymer systems: *In vitro* and *in vivo* evaluation Int. J. Pharm 2008 352 58 65 18036752 10.1016/j.ijpharm.2007.10.016

[113] Na DH Lee KC DeLuca PP PEGylation of octreotide: II. Effect of N-terminal mono-PEGylation on biological activity and pharmacokinetics Pharm. Res 2005 22 743 749 15906169 10.1007/s11095-005-2590-y

[114] Brache V Faundes A Alvarez F Garcia AG Transition from Norplant to Jadelle in a clinic with extensive experience providing contraceptive implants Contraception 2006 73 364 367 16531168 10.1016/j.contraception.2005.10.015

[115] Dasaratha DM Vema K Jayakumar R Vamsadhara C Preparation and characterization of injectable microspheres of contraceptive hormones Int. J. Pharm 2003 268 23 29 14643973 10.1016/j.ijpharm.2003.08.011

[116] Dhanaraju MD Rajkannan R Selvaraj D Jayakumar R Vamsadhara C Biodegradation and biocompatibility of contraceptive-steroid-loaded poly (DL-lactide-co-glycolide) injectable microspheres: *in vitro* and *in vivo* study Contraception 2006 74 148 156 16860053 10.1016/j.contraception.2006.01.015

[117] Pai SS Tilton RD Przybycien TM Poly(ethylene glycol)-modified proteins: implications for poly(lactide-co-glycolide)-based microsphere delivery AAPS J 2009 11 88 98 19199044 10.1208/s12248-009-9081-8 PMC2664882

[118] Wang SH Zhang LC Lin F Sa XY Zuo JB Shao QX Chen GS Zeng S Controlled release of levonorgestrel from biodegradable poly(D,L-lactide-co-glycolide) microspheres: *in vitro* and *in vivo* studies Int. J. Pharm 2005 301 217 225 16040213 10.1016/j.ijpharm.2005.05.038

[119] Cheng YH Illum L Davis SS Schizophrenia and drug delivery systems J. Drug. Targeting 2000 8 107 117 10.3109/10611860008996856 10852342

[120] Hans ML Maxwell C Ehrlichman RS Metzger K Liang Y Siegel SJ Lowman AM Evaluation of *in vitro* release and *in vivo* efficacy of mPEG-PLA-haloperidol conjugate micelle-like structures J. Biomed. Mater. Res. Part B 2007 83 422 430 10.1002/jbm.b.30812 17415770

[121] Lu Y Tang X Cui Y Zhang Y Qin F Lu X *In vivo* evaluation of risperidone-SAIB *in situ* system as a sustained release delivery system in rats Eur. J. Pharm. Biopharm 2008 68 422 429 17614267 10.1016/j.ejpb.2007.05.016

[122] Lu Y Yu Y Tang X Sucrose acetate isobutyrate as an *in situ* forming system for sustained risperidone release J. Pharm. Sci 2007 96 3252 3262 17721936 10.1002/jps.21091

[123] Agnihotri SA Aminabhavi TM Controlled release of clozapine through chitosan microparticles prepared by a novel method J. Control. Release 2004 96 245 259 15081216 10.1016/j.jconrel.2004.01.025

[124] Nahata T Saini TR Optimization of formulation variables for the development of long acting microsphere based depot injection of olanzapine J. Microencapsul 2008 25 426 433 18608793 10.1080/02652040802033913

[125] Guthmann C Lipp R Wagner T Kranz H Development of a novel osmotically driven drug delivery system for weakly basic drugs Eur. J. Pharm. Biopharm 2008 69 667 674 18226884 10.1016/j.ejpb.2007.12.017

[126] McClelland GA Sutton SC Engle K Zentner GM The solubility-modulated osmotic pump: *in vitro*/*in vivo* release of diltiazem hydrochloride Pharm. Res 1991 8 88 92 2014214 10.1023/a:1015890525495

[127] Mohammadi-Samani S Adrangui M Siahi-Shadbad MR Nokhodchi A An approach to controlled-release dosage form of propranolol hydrochloride Drug Dev. Ind. Pharm 2000 26 91 94 10677815 10.1081/ddc-100100332

[128] Nokhodchi A Momin MN Shokri J Shahsavari M Rashidi PA Factors affecting the release of nifedipine from a swellable elementary osmotic pump Drug Delivery 2008 15 43 48 18197523 10.1080/10717540701829028

[129] Wang X Nie SF Li W Luan L Pan W Studies on bi-layer osmotic pump tablets of water-insoluble allopurinol with large dose: *in vitro* and *in vivo* Drug Dev. Ind. Pharm 2007 33 1024 1029 17891589 10.1080/03639040601179897

[130] He L Gong T Zhao D Zhang ZR Li L A novel controlled porosity osmotic pump system for sodium ferulate Pharmazie 2006 61 1022 1027 17283661

[131] Rani M Mishra B Comparative *in vitro* and *in vivo* evaluation of matrix, osmotic matrix, and osmotic pump tablets for controlled delivery of diclofenac sodium AAPS Pharm. Sci. Technol 2004 5 71 10.1208/pt050471 PMC2750496 15760068

[132] Makhija SN Vavia PR Controlled porosity osmotic pump-based controlled release systems of pseudoephedrine. I. Cellulose acetate as a semipermeable membrane J. Control. Release 2003 89 5 18 12695059 10.1016/s0168-3659(02)00482-0

[133] Kang F Singh J Effect of additives on the release of a model protein from PLGA microspheres AAPS Pharm-Sci-Tech 2001 2 30 10.1007/BF02830570 PMC2784845 14727867

[134] Blanco D Alonso MJ Protein encapsulation and release from poly(lactide-co-glycolide) microspheres: effect of the protein and polymer properties and of the co-encapsulation of surfactants Eur. J. Pharm. Biopharm 1998 45 285 294 9653633 10.1016/s0939-6411(98)00011-3

[135] Sandor M Enscore D Weston P Mathiowitz E Effect of protein molecular weight on release from micron-sized PLGA microspheres J. Control. Release 2001 76 297 311 11578744 10.1016/s0168-3659(01)00446-1

[136] Misra SK Ansari TI Valappil SP Mohn D Philip SE Stark WJ Roy I Knowles JC Salih V Boccaccini AR Poly(3-hydroxybutyrate) multifunctional composite scaffolds for tissue engineering applications Biomaterials 2010 31 2806 2815 20045554 10.1016/j.biomaterials.2009.12.045

[137] Francis L Meng D Knowles JC Roy I Boccaccini AR Multi-functional P(3HB) microsphere/45S5 Bioglass-based composite scaffolds for bone tissue engineering Acta Biomater 2010 6 2773 2786 20056174 10.1016/j.actbio.2009.12.054

[138] Mourino V Boccaccini AR Bone tissue engineering therapeutics: Controlled drug delivery in three-dimensional scaffolds J. R. Soc. Interface 2009 7 209 227 19864265 10.1098/rsif.2009.0379 PMC2842615

[139] Dani BA Raiche AT Puleo DA DeLuca PP A study of the antiresorptive activity of salmon calcitonin microspheres using cultured osteoclastic cells AAPS Pharm. Sci. Technol 2002 3 E21 10.1007/BF02830619 PMC2784050 12916936

[140] Raiche AT Puleo DA Association polymers for modulated release of bioactive proteins IEEE Eng. Med. Biol. Mag 2003 22 35 41 14699934 10.1109/memb.2003.1256270

[141] Jeon JH Thomas MV Puleo DA Bioerodible devices for intermittent release of simvastatin acid Int. J. Pharm 2007 340 6 12 17433584 10.1016/j.ijpharm.2007.03.007 PMC2211564

[142] Jeon JH Piepgrass WT Lin YL Thomas MV Puleo DA Localized intermittent delivery of simvastatin hydroxyacid stimulates bone formation in rats J. Periodontol 2008 79 1457 1464 18672996 10.1902/jop.2008.080004

[143] Jeon JH Puleo DA Alternating release of different bioactive molecules from a complexation polymer system Biomaterials 2008 29 3591 3598 18514812 10.1016/j.biomaterials.2008.05.011 PMC2536711

[144] Mansour HM Rhee YS Wu X Nanomedicine in Pulmonary Delivery Int. J. Nanomed 2009 4 299 319 10.2147/ijn.s4937 PMC2802043 20054434

[145] Barichello JM Morishita M Takayama K Nagai T Encapsulation of hydrophilic and lipophilic drugs in PLGA nanoparticles by the nanoprecipitation method Drug Dev. Ind. Pharm 1999 25 471 476 10194602 10.1081/ddc-100102197

[146] Kumar PS Ramakrishna S Saini TR Diwan PV Influence of microencapsulation method and peptide loading on formulation of poly(lactide-co-glycolide) insulin nanoparticles Pharmazie 2006 61 613 617 16889069

[147] Hafeli UO Magnetically modulated therapeutic systems Int. J. Pharm 2004 277 19 24 15158965 10.1016/j.ijpharm.2003.03.002

[148] Cheng J Teply BA Jeong SY Yim CH Ho D Sherifi I Jon S Farokhzad OC Khademhosseini A Langer RS Magnetically responsive polymeric microparticles for oral delivery of protein drugs Pharm. Res 2006 23 557 564 16388405 10.1007/s11095-005-9444-5

[149] Mundargi RC Babu VR Rangaswamy V Patel P Aminabhavi TM Nano/micro technologies for delivering macromolecular therapeutics using poly(D,L-lactide-co-glycolide) and its derivatives J. Control. Release 2008 125 193 209 18083265 10.1016/j.jconrel.2007.09.013

[150] Florence AT Florence AT Siepmann J Pharmaceutical Aspects of Nanotechnology Modern Pharmaceutics: Volume 2-Applications and Advances Informa Healthcare New York, NY, USA 2009 453 492

[151] Hickey AJ Mansour HM Rathbone MJ Hadgraft J Roberts MS Lane ME Formulation Challenges of Powders for the Delivery of Small Molecular Weight Molecules as Aerosols Modified-Release Drug Delivery Technology Informa Healthcare New York, NY, USA 2008 573 602

[152] York P Kompella UB Shekunov BY Supercritical Fluid Technology for Drug Product Development Informa Healthcare New York, NY, USA 2004

[153] Williams JR Clifford AA al-Saidi SH Supercritical fluids and their applications in biotechnology and related areas Mol. Biotechnol 2002 22 263 286 12448881 10.1385/MB:22:3:263

[154] Mishima K Biodegradable particle formation for drug and gene delivery using supercritical fluid and dense gas Adv. Drug Deliv. Rev 2008 60 411 432 18061302 10.1016/j.addr.2007.02.003

[155] Lee LY Wang CH Smith KA Supercritical antisolvent production of biodegradable micro-and nanoparticles for controlled delivery of paclitaxel J. Control. Release 2008 125 96 106 18054107 10.1016/j.jconrel.2007.10.002

[156] Li W Zhang J Zhang C Feng X Han B Yang G Synthesis of alpha-chymotrypsin/polymer composites by a reverse micelle/gas antisolvent method Colloids Surf. B. Biointerfaces 2007 59 11 15 17532613 10.1016/j.colsurfb.2007.04.011

[157] Steckel H Pichert L Muller BW Influence of process parameters in the ASES process on particle properties of budesonide for pulmonary delivery Eur. J. Pharm. Biopharm 2004 57 507 512 15093600 10.1016/j.ejpb.2004.01.002

[158] Bleich J Muller BW Production of drug loaded microparticles by the use of supercritical gases with the aerosol solvent extraction system (ASES) process J. Microencapsul 1996 13 131 139 8999119 10.3109/02652049609052902

[159] Palakodaty S York P Pritchard J Supercritical fluid processing of materials from aqueous solutions: the application of SEDS to lactose as a model substance Pharm. Res 1998 15 1835 1843 9892466 10.1023/a:1011949805156

[160] Chow AH Tong HH Chattopadhyay P Shekunov BY Particle engineering for pulmonary drug delivery Pharm. Res 2007 24 411 437 17245651 10.1007/s11095-006-9174-3

[161] Dunbar CA Concessio NM Hickey AJ Evaluation of atomizer performance in production of respirable spray-dried particles Pharm. Dev. Technol 1998 3 433 441 9834945 10.3109/10837459809028624

[162] Elversson J Millqvist-Fureby A Alderborn G Elofsson U Droplet and particle size relationship and shell thickness of inhalable lactose particles during spray drying J. Pharm. Sci 2003 92 900 910 12661075 10.1002/jps.10352

[163] Elversson J Millqvist-Fureby A Particle size and density in spray drying-effects of carbohydrate properties J. Pharm. Sci 2005 94 2049 2060 16052553 10.1002/jps.20418

[164] Gilani K Najafabadi AR Barghi M Rafiee-Tehrani M The effect of water to ethanol feed ratio on physical properties and aerosolization behavior of spray dried cromolyn sodium particles J. Pharm. Sci 2005 94 1048 1059 15793812 10.1002/jps.20315

[165] Maa YF Costantino HR Nguyen PA Hsu CC The effect of operating and formulation variables on the morphology of spray-dried protein particles Pharm. Dev. Technol 1997 2 213 223 9552449 10.3109/10837459709031441

[166] Rogers TL Johnston KP Williams RO Solution-based particle formation of pharmaceutical powders by supercritical or compressed fluid CO2 and cryogenic spray-freezing technologies Drug Dev. Ind. Pharm 2001 27 1003 1015 11794803 10.1081/ddc-100108363

[167] Maa YF Prestrelski SJ Biopharmaceutical powders: particle formation and formulation considerations Curr. Pharm. Biotechnol 2000 1 283 302 11469385 10.2174/1389201003378898

[168] Costantino HR Firouzabadian L Hogeland K Wu C Beganski C Carrasquillo KG Cordova M Griebenow K Zale SE Tracy MA Protein spray-freeze drying. Effect of atomization conditions on particle size and stability Pharm. Res 2000 17 1374 1383 11205730 10.1023/a:1007570030368

[169] Yu Z Rogers TL Hu J Johnston KP Williams RO Preparation and characterization of microparticles containing peptide produced by a novel process: spray freezing into liquid Eur. J. Pharm. Biopharm 2002 54 221 228 12191695 10.1016/s0939-6411(02)00050-4

[170] Hu J Johnston KP Williams RO Stable amorphous danazol nanostructured powders with rapid dissolution rates produced by spray freezing into liquid Drug Dev. Ind. Pharm 2004 30 695 704 15491047 10.1081/ddc-120039212

[171] D’Souza SS Faraj JA DeLuca PP A model-dependent approach to correlate accelerated with real-time release from biodegradable microspheres AAPS Pharm-Sci-Tech 2005 6 E553 564 10.1208/pt060470 PMC2750603 16408857

[172] D’Souza SS Selmin F Murty SB Qiu W Thanoo BC DeLuca PP Assessment of fertility in male rats after extended chemical castration with a GnRH antagonist AAPS J 2004 6 94 99 15198511 10.1208/ps060110 PMC2750945

[173] Kane JM Eerdekens M Lindenmayer JP Keith SJ Lesem M Karcher K Long-acting injectable risperidone: efficacy and safety of the first long-acting atypical antipsychotic Am. J. Psychiatry 2003 160 1125 1132 12777271 10.1176/appi.ajp.160.6.1125

[174] Kostanski JW Thanoo BC DeLuca PP Preparation, characterization, and *in vitro* evaluation of 1- and 4-month controlled release orntide PLA and PLGA microspheres Pharm. Dev. Technol 2000 5 585 596 11109259 10.1081/pdt-100102043

[175] Okada H Doken Y Ogawa Y Toguchi H Preparation of three-month depot injectable microspheres of leuprorelin acetate using biodegradable polymers Pharm. Res 1994 11 1143 1147 7971715 10.1023/a:1018936815654

[176] Woo BH Na KH Dani BA Jiang G Thanoo BC DeLuca PP *In vitro* characterization and *in vivo* testosterone suppression of 6-month release poly (D,L-lactide) leuprolide microspheres Pharm. Res 2002 19 546 550 12033393 10.1023/a:1015168301339

[177] Matsumura Y Poly (amino acid) micelle nanocarriers in preclinical and clinical studies Adv. Drug Deliv. Rev 2008 60 899 914 18406004 10.1016/j.addr.2007.11.010

[178] Kanagale P Patel V Venkatesan N Jain M Patel P Misra A Pharmaceutical development of solid dispersion based osmotic drug delivery system for nifedipine Curr. Drug Deliv 2008 5 306 311 18855601 10.2174/156720108785914998

